# Endoscopic sleeve gastroplasty: results from a single surgical bariatric centre

**DOI:** 10.1007/s13304-022-01385-4

**Published:** 2022-09-27

**Authors:** Lino Polese, Luca Prevedello, Amanda Belluzzi, Emilia Giugliano, Alice Albanese, Mirto Foletto

**Affiliations:** 1 1St Surgical Unit, Department of Surgery, Oncology and Gastroenterology, Chirurgia Generale 1, sesto piano Policlinico, Azienda Ospedale-Università Padova University of Padova, Via Giustiniani 2, 35128 Padua, Italy; 2 Bariatric Unit, Week Surgery, Padova University Hospital, University of Padova, Padua, Italy

**Keywords:** Endoscopic sleeve gastroplasty, Obesity, Endosleeve, Bariatric surgery, Sleeve gastrectomy

## Abstract

The aim of this study was to evaluate the safety and efficacy of the endoscopic sleeve gastroplasty (ESG) procedure. Patients ineligible for bariatric surgery due to comorbidities or low Body Mass Index (BMI) were offered ESG. Gastric tubularization was carried out via multiple multi-bite sutures across the greater curvature of the stomach. The patients underwent a water-soluble swallow test on post-operative day 1 (POD-1) to assess gastric emptying and were placed on a soft diet if upper GI tract function was confirmed. From January 2019 to March 2022, 27 patients underwent ESG: 14 for severe obesity with comorbidities, including liver transplant, end-stage kidney disease, severe cardiovascular and respiratory diseases. The mean BMI before treatment was 36 ± 9 kg/m^2^. Two patients (7%) who developed gastric bleeding were successfully treated with packed red blood cells (PRBC) transfusions. After a mean follow-up of 18 months, the percentage of total body weight loss (%TBWL) and the percentage of excess weight (%EWL) were 11 ± 7 and 39 ± 27, respectively. The latter was significantly higher in the patients with an initial BMI < 40 kg/m^2^ (50 vs 22, *p* < 0.05). The patients whose gastric sleeve extended for more than a third of the length of the stomach (*p* < 0.05) had better results. ESG was found to be effective and safe in high-risk surgical patients whose initial BMI was (< 40). Studies characterized by larger number of patients and longer follow-up periods will be able to confirm these results.

## Introduction

It is a well-known fact that obesity is a worldwide “plague” that significantly affects the quality of life, morbidity, and mortality of a large percentage of the population and health costs at large. As the global prevalence of obesity increases, the number of individuals who are either overweight or obese already appears to exceed the 2.1 billion mark [1]. In the light of these statistics, bariatric surgery seems to represent a worthwhile measure to induce weight loss in the effort to improve health and reduce comorbidities such as type 2 diabetes, hypertension, obstructive sleep apnoea, dyslipidaemia often noted in obese patients. Some obese patients are nevertheless considered ineligible for bariatric surgery because they are at high surgical risk due to their comorbidities and/or age. Endoscopic bariatric procedures, which are minimally invasive and can be repeated, if necessary [2], may represent a less risky alternative for that group of patients and for individuals who have a lower body mass index (BMI) (e.g. < 35 kg/m^2^).

One of the most promising endoscopic procedures now being used as a weight-loss measure is endoscopic sleeve gastroplasty (ESG) [3]. ESG uses a suturing device to create a tube or sleeve internally without removing any part of the stomach. The device is used to place full-thickness sutures beginning at the gastric incisura and working towards the gastric fundus, preserving the area of the pyloric antrum and the fundus. The gastroplasty restricts the amount of food required to fill the stomach, leading to satiety and weight loss. The aim of the current study was to analyze the safety and efficacy of ESG in a group of patients considered poor-risk surgical candidates or with low BMIs.

## Materials and methods

Between January 2019 and March 2022 obese patients considered at high risk for surgery and patients with BMI < 35 kg/m^2^ without severe comorbidities were offered the option of ESG. Exclusion criteria for the procedure were: severe gastritis, large (> 5 cm) hiatal hernia, portal hypertensive gastropathy, and an unwillingness to undergo blood transfusion.

### Ethical rule statement

The study was performed in accordance with the ethical standards as laid down in the 1964 Declaration of Helsinki and its later amendments. Patients were operated on after giving written informed consent.

### Procedure

Intubated patients under general anaesthesia with carbon dioxide insufflation underwent ESG. The patients were usually positioned in the left lateral position except for those with specific anaesthesiologic risks (in particular for pulmonary disease) requiring a supine position. A gastroscopy was performed to exclude contraindications. An overtube was positioned under endoscopic guidance. The Overstitch (Apollo Endosurgery, Austin, TX), mounted on a double channel gastroscope (Olympus Optica, Tokyo, Japan), was used to place multiple multi-bite interrupted sutures across the greater curve to reduce the stomach volume from the angulus toward the fundus, which was left untouched. Full-thickness 2–0 polypropylene sutures were placed endoluminally by capturing the stomach wall with a helix. The suture pattern was transverse monolinear. [4] Each suture consisted of four to five bites. The number of plications used depended on the distance between the antrum and the fundus.

Following the procedure, the patients were prescribed proton pump inhibitor (PPI), metoclopramide and ondansetron treatment, as needed. On the first postoperative day (POD1) the patients underwent the water-soluble swallow test to evaluate the digestive integrity and the condition of the stomach. If the test resulted normal they were placed on a soft diet. Regular follow-up examinations were scheduled 3, 6 and 12 months after the procedure. The patients were also contacted by telephone when the study was conducted.

**Fig. 1:**
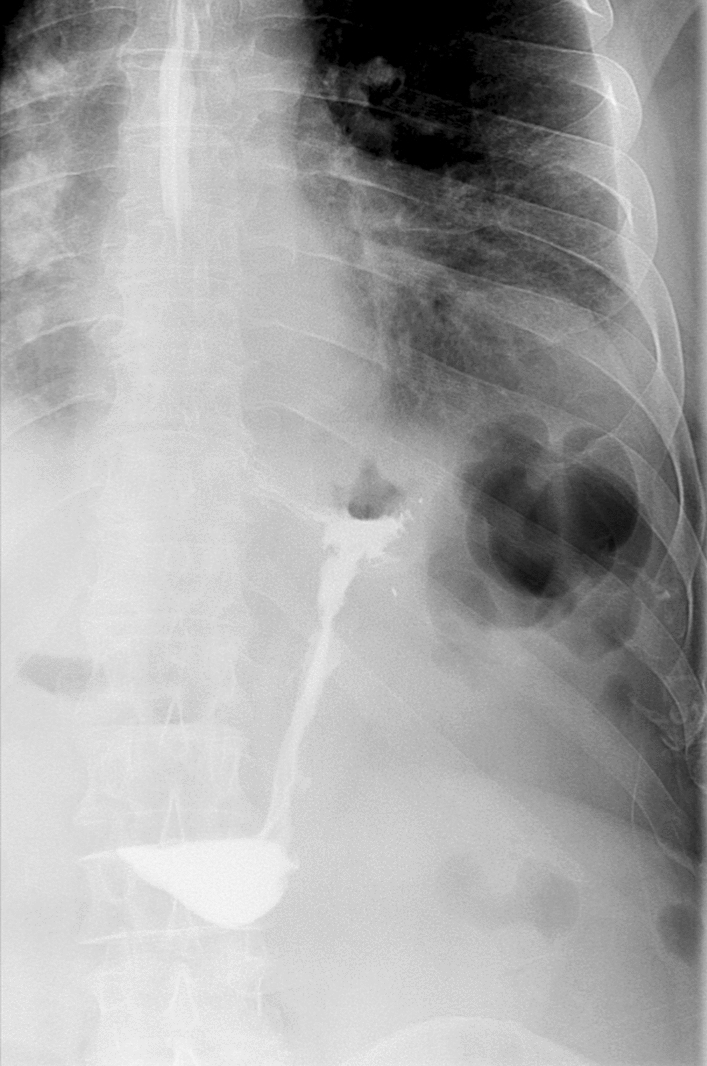
Swallow X-ray after the ESG procedure

The patients’ characteristics, details regarding the ESG procedure including the number of stitches, the percentage of stomach that was tubularized (the length of the stomach tubularized/the total gastric length) and the diameter of the tubularization according to the contrast swallow X-ray were collected. (Fig. 1) The patients’ TBWL and %EWL at each follow-up examination were registered.

### Statistical analysis

The patients’ weights and their %TBWL and %EWL were compared using the *T* test. The relationship between the percentage of tubularized stomach and weight loss was evaluated using the X-square test. The patients’ %TBWL > 15 and %EWL > 35 results were considered good in the light of data reported by other studies [5].

## Results

Between January 2019 and March 2022, 27 patients underwent ESG, 14 for severe obesity with high risk comorbidities, including liver transplant (2), end-stage kidney disease (2), severe cardiovascular and respiratory diseases (10). The mean BMI before treatment was 36 ± 9 kg/m^2^. The mean duration of the ESG procedure was 61 min (range 35–120 min), the number of sutures ranged between 3 and 7 (median 4).

Post-operative complications were reported in 2 of the patients (7%). In one, who had end-stage kidney disease, some oesophageal mucosal tearing took place while the overtube was being inserted. Positioning the overtube in the patient (who had previously undergone thyroid surgery) proved, in fact, to be difficult, and the laceration became evident when the overtube was removed at the end of the procedure. The tear was treated by positioning a nasogastric tube which left in place for 3 days. That patient also presented gastric bleeding, which was successfully treated with a Packed red blood cells PRBC transfusions. Another patient, who was taking antiaggregant drugs, presented post-operative bleeding. He was successfully treated with PRBC transfusions. A post-procedural upper GI endoscopy was not required in either case.

After a mean follow-up of 18 months, the patients’ %TBWL and %EWL were 11 ± 7 and 39 ± 27, respectively (*p* < 0.05). The %EWL was significantly higher in the patients whose initial BMI was < 40 kg/m^2^ (50 vs 22, *p* < 0.05). (Table 1) The number of sutures used did not significantly affect the outcomes. The best results (%EWL > 35) were obtained in the patients who had more than 1/3 of the stomach length tubularized (*p* < 0.05).

**Table 1:** An analysis of predictive factors for endoscopic sleeve gastrectomy

	%EWL	*P*
Initial BMI (< 40 vs > 40)	51 ± 26 vs 23 ± 12	< 0.01
Number stitches (< 4 vs ≥ 4)	50 ± 30 vs 34 ± 22	n.s
Sleeve diameter (< 1 cm vs > 1 cm)	43 ± 22 vs 39 ± 30	n.s
Sleeve length (> 1/3 vs < 1/3 whole stomach)	45 ± 26 vs 18 ± 11	< 0.05

**Fig. 2:**
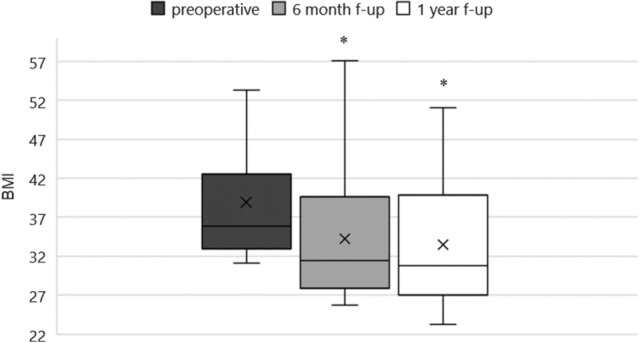
BMI levels preoperatively, 6 months, and 12 months after endoscopic sleeve gastrectomy procedure. *p < 0.01

We have follow-up data regarding at least a year’s time for twenty of the patients; we have follow-up information for longer than 6 months but less than 1 year for 5 patients. One patient was lost to follow-up after undergoing the first scheduled examination, and another died of end-stage kidney disease during the follow-up. Of the 20 patients with more than a 1-year follow-up, weight loss was maintained in 16 (80%), two underwent sleeve gastrectomy and mini gastric bypass, respectively, when the results of the ESG proved unsatisfactory, and 2 regained weight later on. Five patients reported further weight loss one year after the procedure (Fig. 2).

## Discussion

The results of this study confirm that ESG is a safe and effective weight-loss measure in obese patients with high-risk comorbidities, including transplanted patients, patients on dialysis for end stage kidney disease and patients with severe heart disease.

The TBWL of 11 ± 7% found in our patients was inferior to a result (14.8 ± 8.5%) reported by a large prospective observational study on 1000 consecutive patients with a mean BMI 33.3 ± 4.5 kg/m^2^. ^5^Incidentally, the present study was performed during the COVID-19 pandemic, a time during which the lockdown and social restrictions had negative impacts on eating behaviours, significantly affecting weight loss. The effect of the COVID-19 pandemic has been discussed elsewhere [6]. Unhealthy eating patterns and worse weight outcomes were also noted in post-bariatric surgery patients during the COVID-19 emergency [7]. Moreover emotional instability linked to the pandemic seems to have increased the risk of developing dysfunctional eating patterns. [8]

According to some authors, neither the suture pattern nor the number of sutures used seems to influence the outcomes of ESG. An analysis of our data uncovered, however, a correlation between the %EWL and the amount of tubularized stomach. The current study is the first to demonstrate that the length of the tubularization significantly impacts the procedure’s results.

According to systematic review by Due-Petersson et al. [9], ESG led to a significantly greater weight loss and lower rate of adverse events with respect to intragastric balloon insertion. Those authors also pointed out that the intragastric balloon seemed to have a temporary effect lasting as long as the balloon was kept in place. The results of our study, instead, showed that the patients who achieved a satisfactory weight loss also maintained acceptable results in most cases (80%) even over a relatively long period (> 1-year follow-up).

ESG is less effective than laparoscopic sleeve gastrectomy (LSG), but significantly safer. In fact according to a systematic review, there was a 5.2% rate of adverse events linked to ESG with respect to 16.9% linked to LSG (*p* < 0.05). [10] Post-operative complications consisting of two cases of bleeding were noted in 7% of our patients. Both occurred in patients at higher risk of bleeding risks (an antiaggregant user in one case and an individual undergoing dialysis in the other). PRBC transfusions were used successfully in both cases and neither patient required surgical or endoscopic treatments.

Just as the possibility of undergoing ESG was proposed to patients with severe comorbidities, it was also offered to individuals with various degrees of obesity who were ineligible for conventional bariatric surgery. Even those with severe obesity showed a significant weight loss when compliance with the dietary regimen was combined with a sufficiently long tubularization of the stomach (at least a third of the stomach’s length).

According to literature, ESG proved to be more effective with respect to other restrictive endoscopic bariatric procedures. When Khan et al. [1] compared ESG with primary obesity surgery endoluminal (POSE) in a systematic review, they found that the weighted mean difference of %EWL between the two procedures was 6.17 at 6 months and 7.84 at 12 months in favour of ESG (respectively p = 0.01 and 0.06). Similar results were reported in a systematic review published by Gys et al. [11] examining eight clinical trials focussing on ESG. The authors reported that the percentage weight loss (%EWL) at 6 and 12 months was significantly superior for the patients who underwent ESG with respect to POSE.

In conclusion, ESG was found to be safe and effective, especially in patients with an initial BMI of < 40 kg/m^2^ and in those in whom the tubularization extended for more than one-third of the length of the stomach. It also proved to be safe and effective in the high-risk surgical patients. Further studies examining larger numbers of patients and designed with longer follow-up times are warranted to confirm our results.
